# Efficacy and safety of closed-loop control system for type one diabetes in adolescents a meta analysis

**DOI:** 10.1038/s41598-023-40423-y

**Published:** 2023-08-13

**Authors:** Mosleh Jabari

**Affiliations:** Department of Pediatrics, Imam Mohammed Ibn Saud Islamic University, An Nada, 13317 Riyadh, Saudi Arabia

**Keywords:** Endocrinology, Health care

## Abstract

This meta-analysis compares the efficacy and safety of Closed-Loop Control (CLC) to Sensor-Augmented Insulin Pump (SAP) for adolescent patients with Type 1 Diabetes Mellitus (T1DM). Eleven randomized-controlled trials were included with a total of 570 patients, from a total of 869 articles found adhering to PRISMA guidelines. The efficacy of the therapies were evaluated from the day, night and during physical activities monitoring of the of the mean blood glucose (BG), Time In Range (TIR), and Standard Deviation (SD) of the glucose variability. The safety measure of the therapies, was assessed from the day and night recording of the hypoglycemic and hyperglycemic events occurred. Pooled results of comparison of mean BG values for day, night and physical activities, − 4.33 [− 6.70, − 1.96] (P = 0.0003), − 16.61 [− 31.68, − 1.54] (P = 0.03) and − 8.27 [− 19.52, 2.99] (P = 0.15). The monitoring for day, night and physical activities for TIR − 13.18 [− 19.18, − 7.17] (P < 0.0001), − 15.36 [− 26.81, − 3.92] (P = 0.009) and − 7.39 [− 17.65, 2.87] (P = 0.16). The day and night results of SD of glucose variability was − 0.40 [− 0.79, − 0.00] (P = 0.05) and − 0.86 [− 2.67, 0.95] (P = 0.35). These values shows the superiority of CLC system in terms of efficacy. The safety evaluation, of the day, night and physical activities observations of average blood glucose goal hypoglycemic events − 0.54 [− 1.86, 0.79] (P = 0.43), 0.04 [− 0.20, 0.27] (P = 0.77) and 0.00 [− 0.25, 0.25] (P = 1.00) and hyperglycemic events − 0.04 [− 0.20, 0.27] (P = 0.77), − 7.11 [− 12.77, − 1.45] (P = 0.01) and − 0.00 [− 0.10, 0.10] (P = 0.97), highlights the commendable safety factor of CLC. The CLC systems can be considered as an ideal preference in the management of adolescents with type 1 diabetes to be used during a 24 h basis.

## Introduction

Children coping with diabetes mellitus (DM) has been a crisis globally, which is a cluster of metabolic diseases, rather than a single illness, that are characterized by chronic hyperglycemia^1^. Type 1 Diabetes Mellitus (T1DM), an autoimmune disease destructs the pancreatic islet cells due to the inability in producing insulin, hormone essential for metabolism of glucose^2^. The reported cases of children with T1DM is rising staggeringly, and about 96,000 children under the age of fifteen are diagnosed with T1DM annually^3^. There are variability in the prevalence of adolescent T1DM with the highest incidence of T1DM reported in the United States (US), India and Brazil^4^. There is a huge economic burden from the chronic nature of the disease due to its multiple short and long-term complications, presenting it as a global health crisis to handle^5^.

Due to β-cell destruction and absolute insulin deficiency nature of T1DM^6^, the therapeutic goal for adolescents with type 1 diabetes is to reach an optimal glycemic control to avoid acute and chronic complications without compromising the wellbeing of children, and social outcomes their families^7^ The complicated nature of management of adolescent T1DM is aimed to prevent the development of complications such as cardiovascular disease, retinopathy, nephropathy, and neuropathy by achieving optimal glycaemia, avoiding hypoglycemic events^8^.

Glycemic targets for children with T1DM have become more rigorous over time, and the average blood glucose (sugar) level goal is now 70 to 120 mg/dL (4 to 7 mmol/L) for all children regardless of age^9,10^. The handling of hypoglycemic remains a challenge with adolescents due to their varied physical activities and requirements. Only less than 70% the youth affected with T1DM achieve glycemic targets despite advances in insulin therapies, educational awareness, insulin pumps, and ‘continuous glucose monitoring systems^11^. The daily tasks of dietary intake control, strict medical control and continuous monitoring makes it difficult for young youth to control their T1DM situations.

The management of T1DM has been revolutionized by the recent advances in technology, like the insulin pumps and continuous glucose monitoring sensors^12^. The Sensor-Augmented Insulin Pump (SAP), which mimics the insulin production by the pancreas via continuous subcutaneous insulin injection (CSII), is a conventional treatment^13^. This traditional approach of SAP therapy does not functionally prevent the chances of occurrence of hypoglycemia. This was a hindrance to the guarantee that the mean blood glucose value will meet therapeutic expectations after using the SAP therapy^14^. The most current development is the integration of the insulin pumps and continuous glucose monitoring sensors, to modify and administer insulin based on the values detected by the sensor^15^. This paved way for the artificial pancreas or called as the closed-loop system for diabetes management. The Closed-Loop Control System (CLC) insulin delivery systems is characterized by real-time glucose-responsive insulin administration and combines glucose-sensing and insulin- delivery components^16^.

Though these advances in diabetes technology are widely used in clinical practice^17^, clinical evidence for the practice of use of CLC insulin delivery as an alternative to SAP therapy has not yet done or available^18^. Studies on inpatient and outpatient adolescent patients with T1DM have been done on closed-loop systems with improved glycemic outcomes and reduction in hypoglycemia in children, especially in the overnight period. CLC has been associated with fewer adverse effects than other insulin therapies in the treatment of adolescents with T1DM^19–26^. Though the effectiveness SAP insulin delivery was stated to be effective in maintaining the daytime glycemic outcomes, it needs to be compared with contemporary interventions^27–29^. There is a serious gap in the availability of evidence to support clinical decisions for the right insulin therapy for adolescent T1DM. It is very crucial to identify if Closed-loop insulin delivery can be a potential replacement for SAP insulin therapy of adolescent T1DM management by comparing the glycemic outcomes and hypoglycemic events to measure the adverse effects of the therapies. This meta-analysis is therefore aimed to compare the efficacy and safety of CLC to SAP for adolescents with type 1 diabetes (T1DM) by evaluating the glycemic outcomes and hypoglycemic events of the two therapies.

The participants, intervention, comparisons, outcomes, and type of studies (PICO) of the current review were as follows: Participants (P): Patients younger than 19 years, Intervention (I): Closed-Loop Control (CLC) in T1DM adolescent patients. Comparisons (C): Sensor-augmented insulin pump (SAP) in T1DM adolescent patients. Outcomes (O): Efficacy of the intervention assessed by average blood glucose value measured by continuous glucose measurement, time in range (TIR), and standard deviation (SD) of glucose variability. Safety was evaluated according to reported hypoglycemic events. Hence this review raises the research question “Does Closed-Loop Control System have a better efficacy and safety compared to Sensor-Augmented Insulin Pump for managing type one diabetes in adolescents?”

## Methodology

This study adheres to The Preferred Reporting Items for Systematic Reviews and Meta-Analyses (PRISMA) guidelines^30^ and complies all the steps advised in Cochrane handbook of systematic reviews. This study has been registered with Prospero with ID CRD42022333310.

### Search strategy

A through bibliographic search of electronic databases of PubMed, MEDLINE, EMBASE, and the Cochrane Library was undertaken in this study. Boolean operators were used for the keywords designed, for searching and identifying the relevant literature. Table 1 is explanatory of the combination of keywords used in this study for the assimilation of articles to review. Grey literature was obtained by searching Web of Science, ProQuest Dissertations and clinicaltrials.gov. The reference sections of retrieved original articles and reviews were scanned for studies that might have been missed in the primary searches. Studies were filtered with regard to study design, methodological features, the reported glycemic outcomes, and the adverse effects evaluated under each study.

**Table 1:** Keyword strategy used in the database search.

Database	Keyword strategy
PubMed, Web of science, Cochrane, and others	1. Adolescent type 1 diabetes mellitus **AND** Insulin therapies **OR** Sensor pumps
	2. Pediatric type 1 diabetes mellitus **AND** Sensor-augmented insulin pump **AND** Closed loop control
	3. Adolescent type 1 diabetes mellitus **AND** Closed loop control OR sensor-augmented insulin pump **NOT** Adults, non-English

The participants, intervention, comparisons, and outcomes (PICO) of the current meta-analysis were as follows.

Participants (P): Patients younger than 19 years as per the definition of an adolescent as a person “10 to 19 years inclusive” and a child “a person 19 years or younger”^31^. Intervention (I): Closed-Loop Control (CLC) in T1DM adolescent patients. Comparisons (C): Sensor-Augmented Insulin Pump (SAP) in T1DM adolescent patients. Outcomes (O): Efficacy of the intervention assessed by average blood glucose value measured by continuous glucose measurement, time in range (TIR), and standard deviation (SD) of glucose variability. Safety was evaluated according to reported hypoglycemic events.

### Data extraction

**Figure 1:**
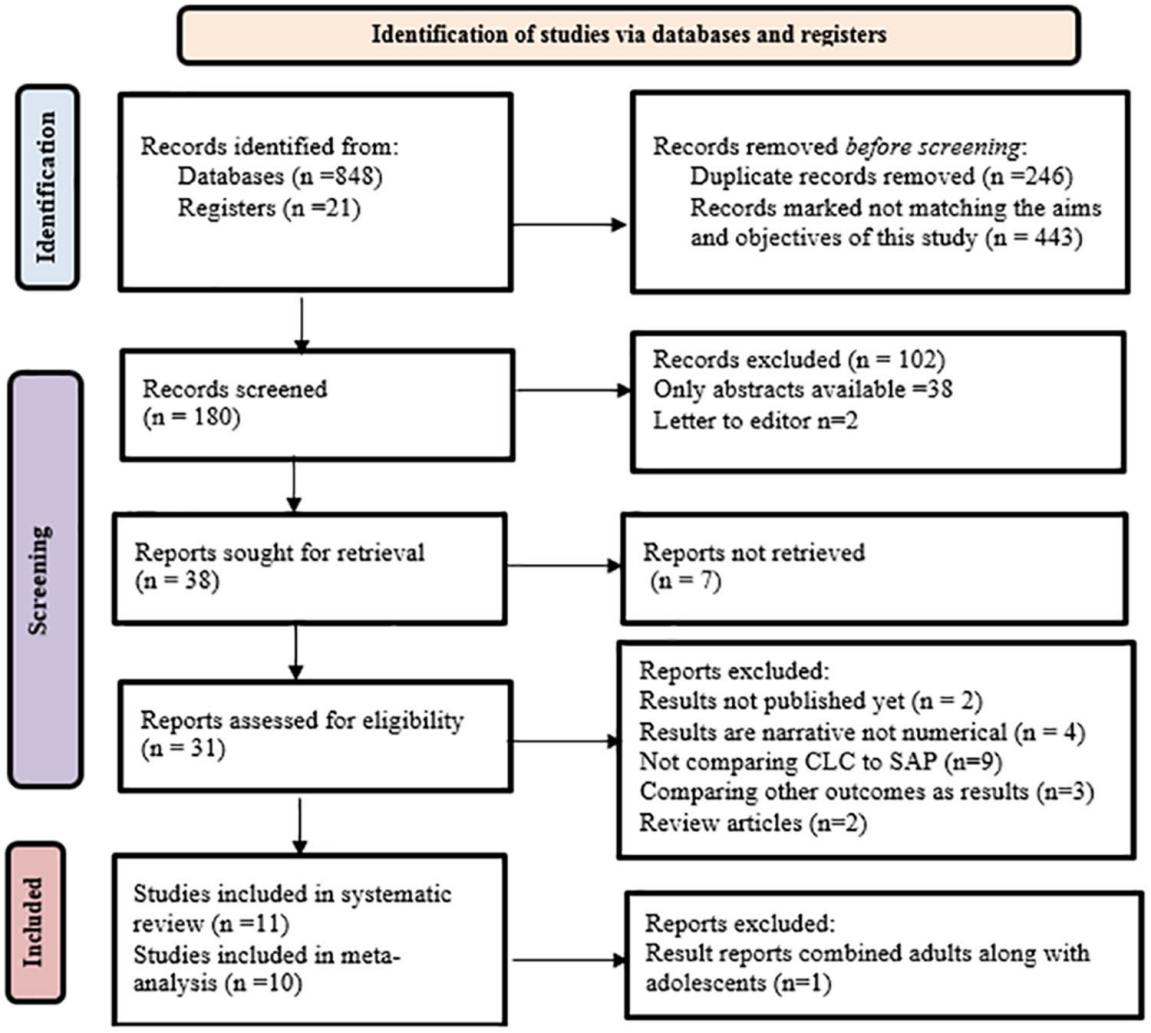
PRISMA flow chart descriptive of the study selection^30,32^.

Relevant full text articles were assimilated after review of the titles and abstracts. The eligibility criteria including the inclusion and exclusion criteria for this study are described in Table 2. Figure 1 illustrates the PRISMA flow diagram for the studies selected in the search process and eligibility appraisal. Review manager 5.4.1 (Revman, Cochrane Collaboration, and Oxford, UK) was used to manage, analyze, and synthesize the included study data. The institutional research board and ethics committee ruled out that approval was not required for this study being a review study.

**Table 2:** Eligibility criteria for study selection in this study.

Inclusion criteria	Exclusion criteria
Experimental studies from 2015 till 2022, comparing Closed-Loop Control (CLC) to sensor-augmented insulin pump (SAP)	Animal studies
Studies which included at least 3 of the glycemic outcomes: % time in range (TIR) 70–180 mg/dL, mean glucose, mg/dL, coefficient of variation and hypoglycemic events	Reviews, letters, editorials, survey reports, abstracts only available
Studies participating T1DM adolescent patients	Studies with descriptive results and outcomes not numerically reported
Full text availability	Non-English articles

Data extraction of the related was done using a custom made data-extraction form in excel. All relevant information on the included studies was extracted into an electronic database, including participant and intervention characteristics, relevant glycemic outcomes and hypoglycemic events to evaluate the adverse effects of treatments, type of insulin delivery technology used and industrial funding or influence on the study.

### Quality assessment

**Figure 2:**
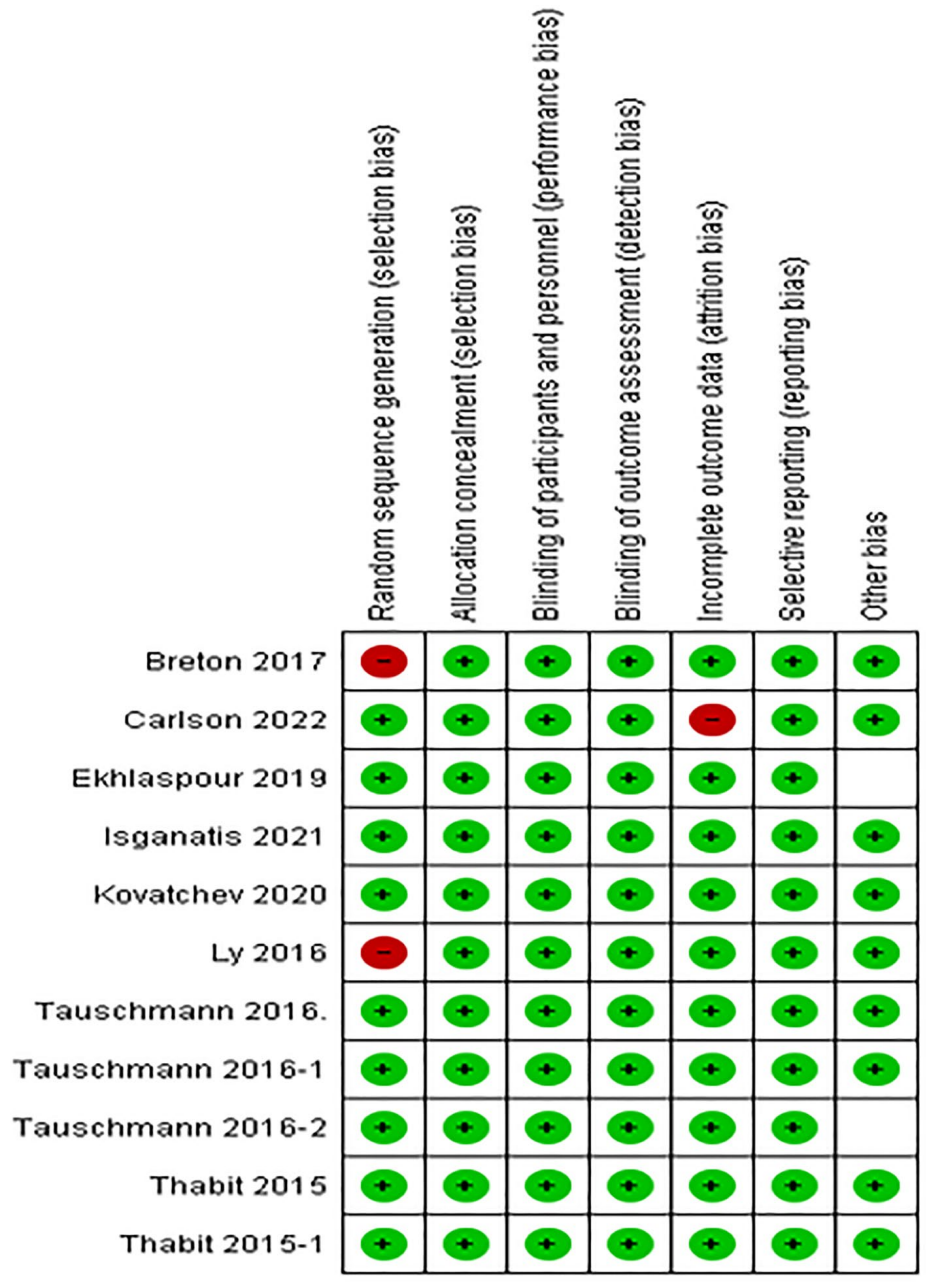
Risk of bias summary about the methodological quality of studies included using the Cochrane risk of bias tool. Symbols show low risk of bias (+), and high risk of bias (−).

The risk of bias method from the Cochrane Collaboration was used^33^ to appraise the quality of the included studies. The studies were graded as low, high, or unclear risk of bias for each of the following items using this method. The domains included in this grading of risk of bias were the random sequence generation and allocation concealment, (both items relate to selection bias), masking of participants and personnel (detection bias), incomplete outcome data (attrition bias), selective reporting (reporting bias), and other biases. Figure 2 is descriptive of the risk of bias tool used in this study.

### Outcome evaluation

#### Efficacy: glycemic outcomes-day, night and during strenuous physical activities

The primary endpoints were the day, night and during strenuous physical activities monitoring of the mean (1) Blood Glucose (BG) level from continuous glucose monitoring, (2) Time In range (TIR) for the percentage of time spent in normoglycemia, 70–180 mg/dL^34^, and (3) Standard Deviation (SD) of glucose variability.

#### Safety: adverse effects-day, night and during strenuous physical activities

The adverse effects outcomes were analyzed from the day, night and during strenuous physical activities monitoring of the time spent while in hypoglycemia < 70 mg/dL, and in hyperglycemia > 250 mg/dL^34^.

### Data analysis

Statistical analysis and the assessment of heterogeneity was done for each reported outcomes in the included studies. All the aggregated outcome measurements has been unified in units to meaningfully analyze the data. The weighted mean difference (WMD) with 95 % confidence interval (CI) was calculated for all the continuous outcomes. The *I*^2^ statistic and χ^2^ test was used to evaluate the heterogeneity of the analysis results. If *I*^2^ > 50% or *p* < 0.1 for the χ^2^ test, the random-effects model was adopted; otherwise, the fixed- effects model was used^35^. P value less than 0.05 was considered statistically significant. All statistical analyses were performed by RevMan software (version 5.0, Oxford, United Kingdom). When the level of heterogeneity was less than 50%, a fixed-effect model was used^36^. The meta-analysis was performed with Review manager 5.4.1 (RevMan, Cochrane Collaboration, Oxford, UK).

### Informed consent

For this type of study, formal consent is not required.

### Patient and public involvement

This study being a meta-analysis systematic literature review, the patients selected were recruited by the researchers of the included studies. All the patient and families related aspects involved in design and implementation of the interventions were priori addressed by the authors of the selected studies.

## Results

### Study search and data extraction

An initial search gave 869 articles from the keyword combinations. The included trials were published between 2015 and 2022. After strict screening and quality assessment 11^37–47^ Randomized Controlled Trials (RCT) were selected for the review which were relevant to the search terms and criteria. A total of 570 adolescent patients were included in this study from the selected studies. This included 298 patients who took CLC insulin therapy and 272 patients who had SAP as their insulin therapy. Only 10^37–44,46,47^ articles were included in the meta-analysis after reviewing and accounting for heterogeneity. (Fig. 1). Table 3 provides the summary of the data extracted from the attributes of the included studies.

**Table 3:** The summary of the attributes included studies.

Study	Study type	Setting	Follow up	No of patients			Day	Night	With Exercise	Therapeutic systems used		Remarks	Industrial funding
				CLC	SAP	#				CLC	SAP		
Carlson 2020^38^	RCT	Multicenter Outpatient, USA	90 days	39	39	39	Y	Y	N	DreaMed Diabetes	MiniMed 670G insulin pump with Guardian Sensor and Guardian Link transmitter	CLC can achieve the recommended glycemic targets in adolescents with T1DM and had improved user experience	Supported by DreaMed Diabetes
Isganaitis 2021^40^	RCT	Multicenter Outpatient, USA	6 months	40	23	63	Y	N	N	Tandem Control-IQ	Dexcom G6 CGM	CLC shown to have the potential to improve glycemic outcomes	Partially supported by Tandem Diabetes Care
Kovatchev 2021^41^	RCT	Multicenter Outpatient, USA	3 months	63	61	124	Y	N	N	Accu-Chek Spirit Combo insulin pump .Dexcom G4 with Control APsoftware	Dexcom (CGM sensors), Roche Diabetes Care (insulin pumps)	CLC is feasible and could offer certain usability advantages over SAP	Partial support from Type Zero Technologies, Dexcom, Roche Diabetes Care and Ascensia Diabetes Care
Ekhlaspour, 2019^39^	RCT	Multicenter, Outpatient, USA	48 h	12	12	24	Y	Y	Y	Tandem t: slim, Insulet Omnipod, Medtronic pumps	Tandem t: slim X2 with Control-IQ Technology	CLC is reported to have improved glycaemic control and safely reduced exposure to hyperglycemia especially during extreme physical activities	Funded by Tandem Diabetes Care
Breton 2017^37^	RCT	Multicenter, Outpatient, USA	6 days	16	16	32	Y	Y	Y	Tandem t:slim, Insulet Omnipod, Medtronic pumps	DiAs Web Monitoring [DWM	CLC in adolescents with T1DM improved glycemic control and reduced exposure to hypoglycemia during prolonged intensive winter sport activities	Partially funded by Dexcom
Ly 2016^42^	RCT	Multicenter, Outpatient, USA	5 days	17	16	33	Y	Y	Y	Dexcom G4 PLATINUM Share glucose sensor and Roche Accu-Chek pump	study CGM and with personal continuous subcutaneous insulin infusion	Increasing time spent in range and reducing both hypoglycaemia and hyperglycemia in adolescents with type 1 diabetes compared with SAP therapy alone	Funded by Tandem Diabetes Care (San Diego, CA), Dexcom
Tauschmann 2016^43^	RCT	Multicenter Multinational, Outpatient. USA and UK	12 weeks	46	40	86	Y	Y	N	Medtronic, Northridge, CA, Satellite 3 glucose sensor (Medtronic), and Contour Next Link 2.4 glucometer	640G insulin pump)	Hybrid closed-loop insulin delivery improves glucose control while reducing the risk of hypoglycaemia	Partially funded by Medtronic, Northridge, CA, USA
Tauschmann 2016 ^44^	RCT	Single centre Outpatient. UK	3 weeks	12	12	12	Y	Y	N	Study insulin pump (DANA Diabecare R)	FlorenceD2A closed-loop system	Improved glucose control during closed-loop along with positive attitudes and experience of the participants with the closed-loop system	Supported by Abbot Diabeted care
Tauschmann 2016^45^	RCT	Single centre Outpatient. UK	7 days	12	12	12	Y	Y	N	Study insulin pump (DANA Diabecare R)	FlorenceD2A closed-loop system	CLC is stated to have a better glucose control without increasing the risk of hypoglycemia in adolescents	Supported by Abbot Diabeted care
Thabit 2015^46^	RCT	Multicenter, Multinational, Outpatient. United Kingdom	12 weeks	25	25	25	Y	Y	N	Insulin pump Abbott Diabetes Care	Abbott Diabetes Care CGM with control algorithm	CLC is stated to show improved glucose control, reduced hypoglycemia in adolescents with T1DM	Supported by Abbot Diabeted care
Thabit 2015^47^	RCT	Multicentre, Multinational, Outpatient. United Kingdom	3 weeks	16	16	16	N	Y	N	Study insulin pump (Dana R Diabecare)	The Florence automated closed-loop system	Overnight CLC at home in adolescents with type 1 diabetes is feasible, showing improvements in glucose control and reducing the risk of nocturnal hypoglycaemia	Supported by Abbot Diabeted care

### Characteristics and quality of trials

In relation to the masking of participants and personnel, almost all of the trials were rated at ‘‘low risk of bias’’ (9 of 11 trials, 81.81%); as for attrition bias and reporting bias, almost all the trials were rated at ‘‘low risk of bias,’’ because they reported the complete outcome data (10 out of 11 trials, 90.90%). There were no studies at ‘‘high risk of bias’’ with any issues relating to random sequence generation, allocation concealment and masking of outcome assessment (11 out of 11 trials, 100%). Figure 2 shows the risk of bias summary based on review quality appraisal judgements about each risk of bias item for each included study.

### Efficacy: glycemic outcomes during day, night and during strenuous physical activities

The results from the included studies were pooled by unifying the measurement units to mg/dL. Hence all the pooled comparison results in this meta-analysis are in mg/dL.

### Mean blood glucose (BG) level: day, night and during strenuous physical activities

**Figure 3:**
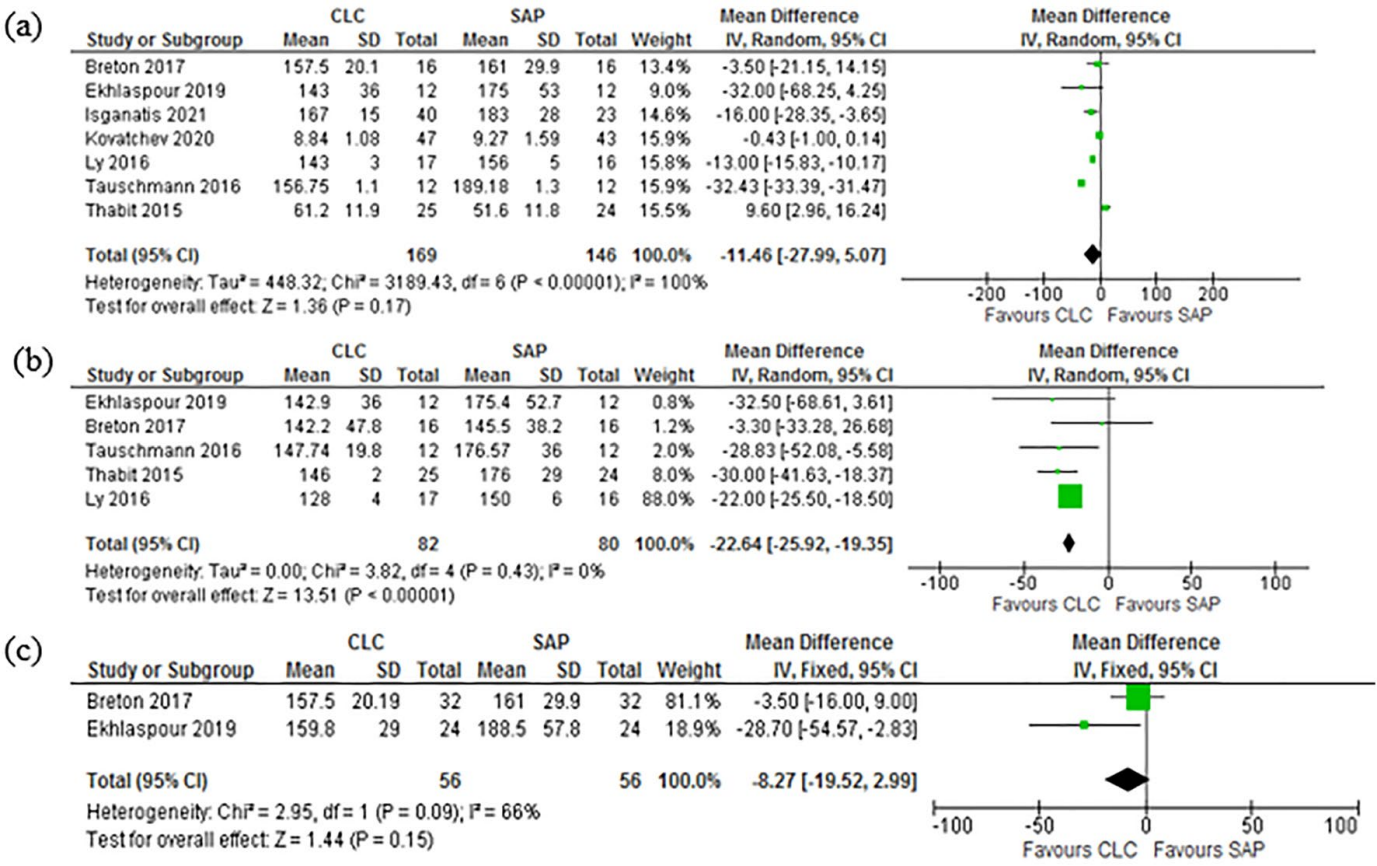
(**a**) Forest plot of comparison: Mean BG-Day. (**b**) Forest plot of comparison: Mean BG-Night. (**c**) Forest plot of comparison: Mean BG-Physical activity.

The average BG was compared in 9 studies^37,39–44,46^. The day monitoring comparison of the BG level showed [Mean Difference (IV, Random, 95% CI) − 4.33 [− 6.70, − 1.96]]. Pooled studies show [Heterogeneity: Tau^2^ = 6.24; Chi^2^ = 77.30, df = 8 (P < 0.00001); I^2^ = 90%. Test for overall effect: Z = 3.58 (P = 0.0003)]. The night monitoring level of BG was reported by 5 studies^37,39,42,46,47^, and it was compared. The results showed (Mean Difference (IV, Random, 95% CI) − 16.61 [− 31.68, − 1.54]). Pooled studies show [Heterogeneity: Tau^2^ = 215.07; Chi^2^ = 75.06, df = 4 (P < 0.00001); I^2^ = 95%. Test for overall effect: Z = 2.16 (P = 0.03)]. The Forest plot in Fig. 3a and b are illustrative of these results. Only two studies^37,39^ showed the results for glycemic outcome during extreme physical activities like winter sports. The physical activity monitoring comparison of the BG level demonstrates [Mean Difference (IV, Random, 95% CI) − 8.27 [− 19.52, 2.99]]. Pooled studies show [Heterogeneity: Chi^2^ = 2.95, df = 1 (P = 0.09); I^2^ = 66%. Test for overall effect: Z = 1.44 (P = 0.15)]. Figure 3c depicts this observation.

### Time in range (TIR): day, night and during strenuous physical activities

**Figure 4:**
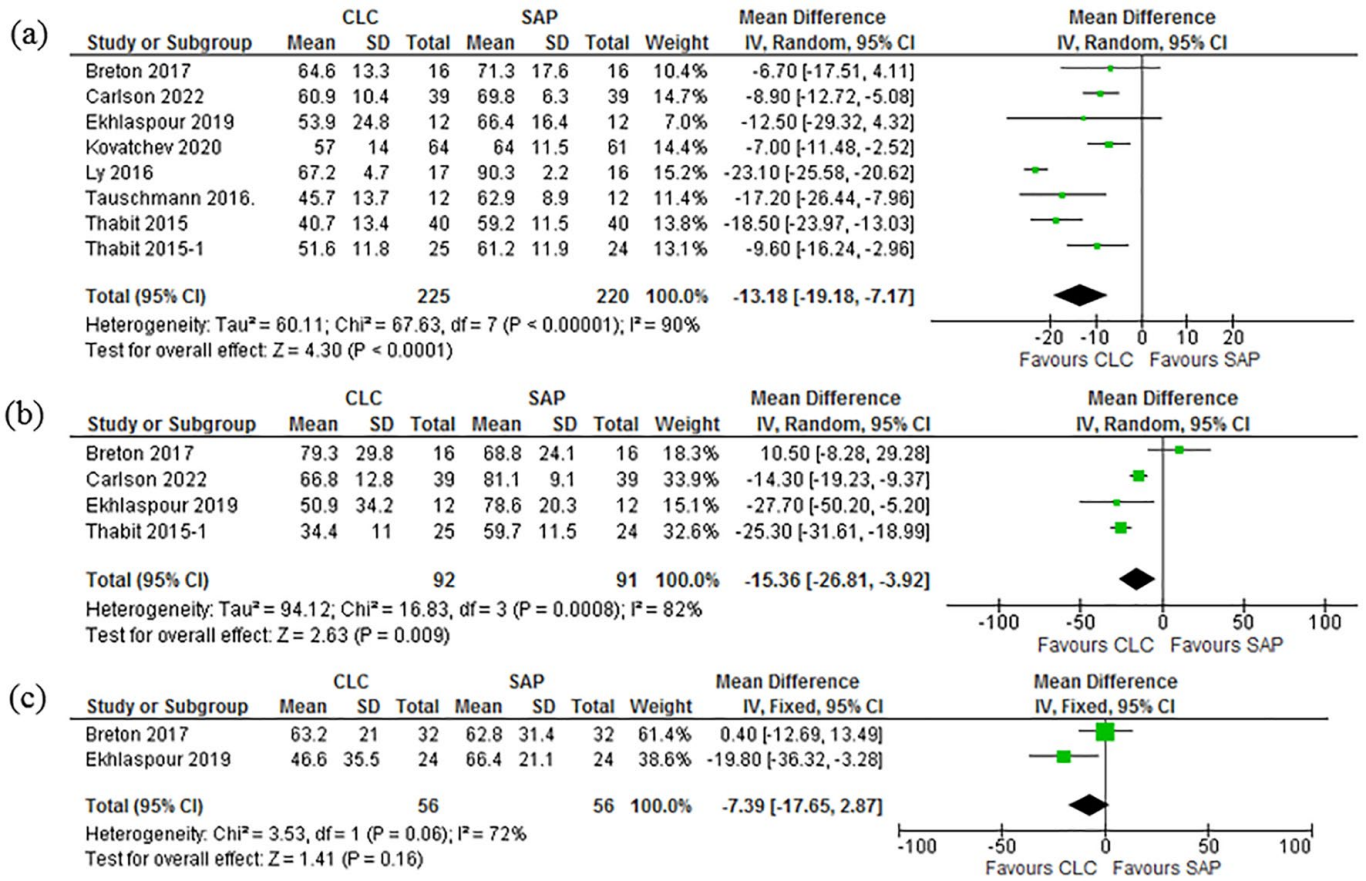
(**a**) Forest plot of comparison: Time in Range TIR-Day. (**b**) Forest plot of comparison: Time in Range TIR-Night. (**c**) Forest plot of comparison: Time in Range TIR-Physical activity.

Time in range (TIR) for the percentage of time spent in normoglycemia, during the day 70–180 mg/dL was compared in all the 10 studies^37–44,46,47^. The results showed (Mean Difference (IV, Random, 95% CI) − 13.18 [− 19.18, − 7.17]. Pooled daytime study aggregate show [Heterogeneity: Tau^2^ = 60.11; Chi^2^ = 67.63, df = 7 (P < 0.00001); I^2^ = 90%. Test for overall effect: Z = 4.30 (P < 0.0001)] Excluding 4 studies^40,43,44,46^ to due to statistical heterogeneity only four studies^37–39,46^ which reported the night time monitoring results of TIR were pooled. The comparison meta results show [Mean Difference (IV, Random, 95% CI) − 15.36 [− 26.81, − 3.92]]. Pooled studies for nighttime data shows [Heterogeneity: Tau^2^ = 94.12; Chi^2^ = 16.83, df = 3 (P = 0.0008); I^2^ = 82%.Test for overall effect: Z = 2.63 (P = 0.009)]. The Forest plot in the Fig. 4a and b elaborates these findings. Two studies^37,39^ recorded the TIR during strenuous physical activities and the results are as follows show [Mean Difference (IV, Random, 95% CI) − 7.39 [− 17.65, 2.87]]. Pooled studies for nighttime data shows [Heterogeneity: Chi^2^ = 3.53, df = 1 (P = 0.06); I^2^ = 72%. Test for overall effect: Z = 1.41 (P = 0.16)]. Figure 4a–c are illustrative of these results.

### Standard deviation (SD) of glucose variability: day and night

**Figure 5:**
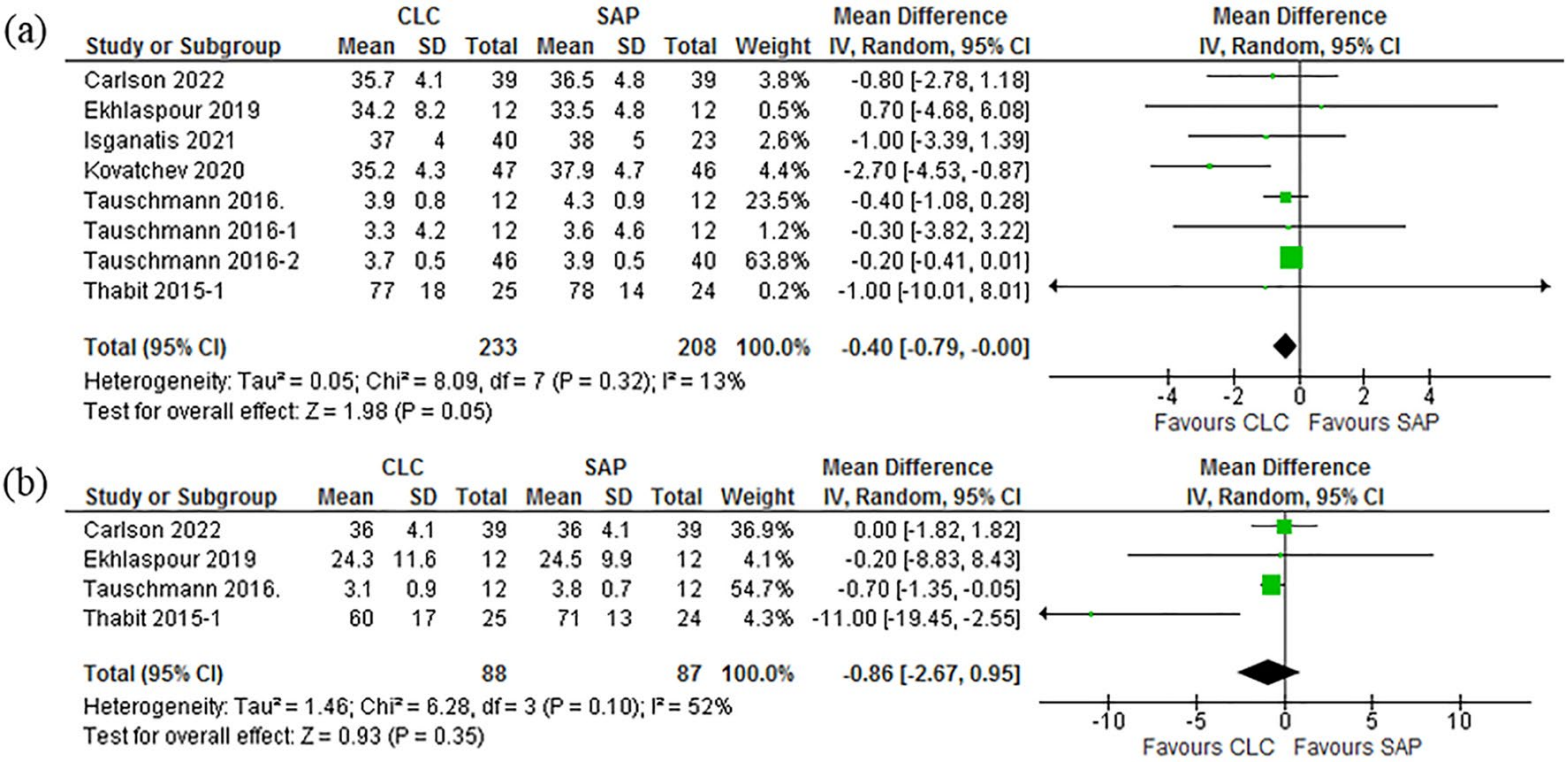
(**a**) Forest plot of comparison: SD of glucose variability-Day. 5(**b**) Forest plot of comparison: SD of glucose variability-Night.

The SD of glucose variability for day time monitoring was compared all the 10 studies^37–44,46,47^ (Mean Difference (IV, Random, 95% CI) − 0.40 [− 0.79, − 0.00]]. Pooled studies for daytime measurements were homogeneous with results as shown [Heterogeneity: Tau^2^ = 0.05; Chi^2^ = 8.09, df = 7 (P = 0.32); I^2^ = 13%. Test for overall effect: Z = 1.98 (P = 0.05)]. The night SD of glucose variability was compared in 5 studies^38,39,43,45,46^ as reported. The results of the pooled studies were (Mean Difference (IV, Random, 95% CI) -0.86 [− 2.67, 0.95] Pooled studies were homogeneous with results as shown [Heterogeneity: Tau^2^ = 1.46; Chi^2^ = 6.28, df = 3 (P = 0.10); I^2^ = 52%. Test for overall effect: Z = 0.93 (P = 0.35)]. Figure 5a and b shows the Forest plot of this stated results. Figure 5a and b are explaining these results. Two studies^39^^,50^ reported the variability as the coefficient of variation and it was converted to standard deviation and pooled. It can be noted that no studies directly reported the standard deviation of glucose variability during physical activities.

### Safety: adverse effects (AE) outcomes

#### Hypoglycemic events: day, night and during strenuous physical activities

**Figure 6:**
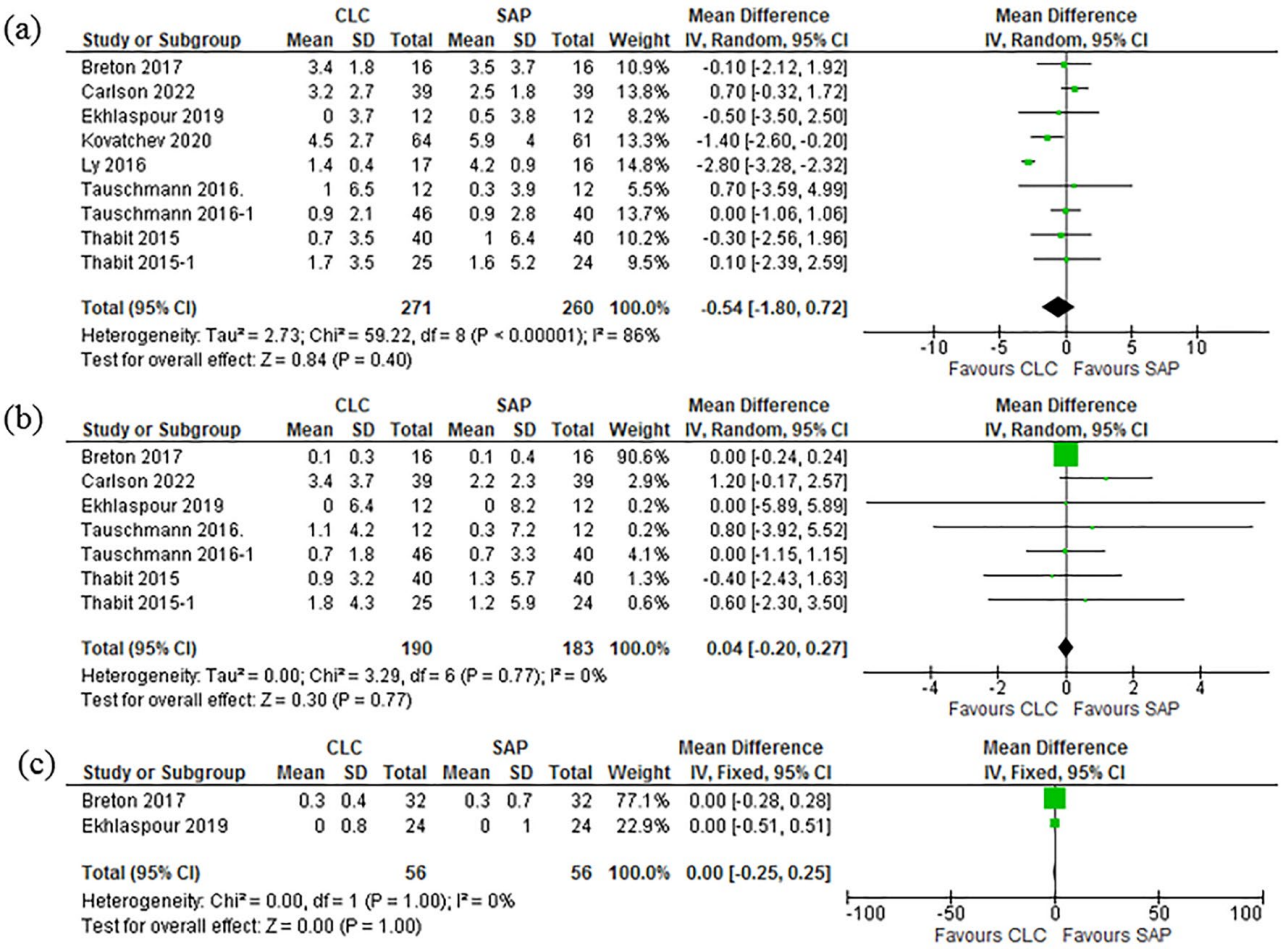
(**a**) Forest plot of comparison: Hypoglycemia-Day. (**b**) Forest plot of comparison: Hypoglycemia-Night. (**c**) Forest plot of comparison: Hypoglycemia-Physical activity.

AEs were compared in 10 studies^37–44,46,47^, including a total of 570 subjects. After excluding studies to account for statistical heterogeneity, the day time reporting results for hypoglycemic events was pooled in from 9^37–39,41–44,46,47^ of the included studies are (Mean Difference (IV, Random, 95% CI) − 0.54 [− 1.86, 0.79]]. Pooled studies exhibits heterogeneity, which is shown as [Heterogeneity: Tau^2^ = 2.78; Chi^2^ = 58.72, df = 8 (P < 0.00001); I^2^ = 86%. Test for overall effect: Test for overall effect: Z = 0.80 (P = 0.43)]. The 7 studies^37–40,43,44,46,47^ were pooled in after accounting for heterogeneity, reported the night comparison results for hypoglycemia and the results shows (Mean Difference (IV, Random, 95% CI) 0.04 [− 0.20, 0.27]). Pooled studies show homogenous results which were, [Heterogeneity: Tau^2^ = 0.00; Chi^2^ = 3.29, df = 6 (P = 0.77); I^2^ = 0%. Test for overall effect: Z = 0.30 (P = 0.77)]. Forest plot in Fig. 6a and b are descriptive of these meta results. The pooled results from the two studies^37,39^ which included results for hypoglycemia during physical activities are as follows, (Mean Difference (IV, Random, 95% CI) 0.00 [− 0.25, 0.25]). Pooled studies show no heterogeneity, which is shown as [Heterogeneity: Chi^2^ = 0.00, df = 1 (P = 1.00); I^2^ = 0%. Test for overall effect: Z = 0.00 (P = 1.00)]. Figure 6a–c are descriptive of these results.

#### Hyperglycemic events: day, night and during strenuous physical activities

**Figure 7:**
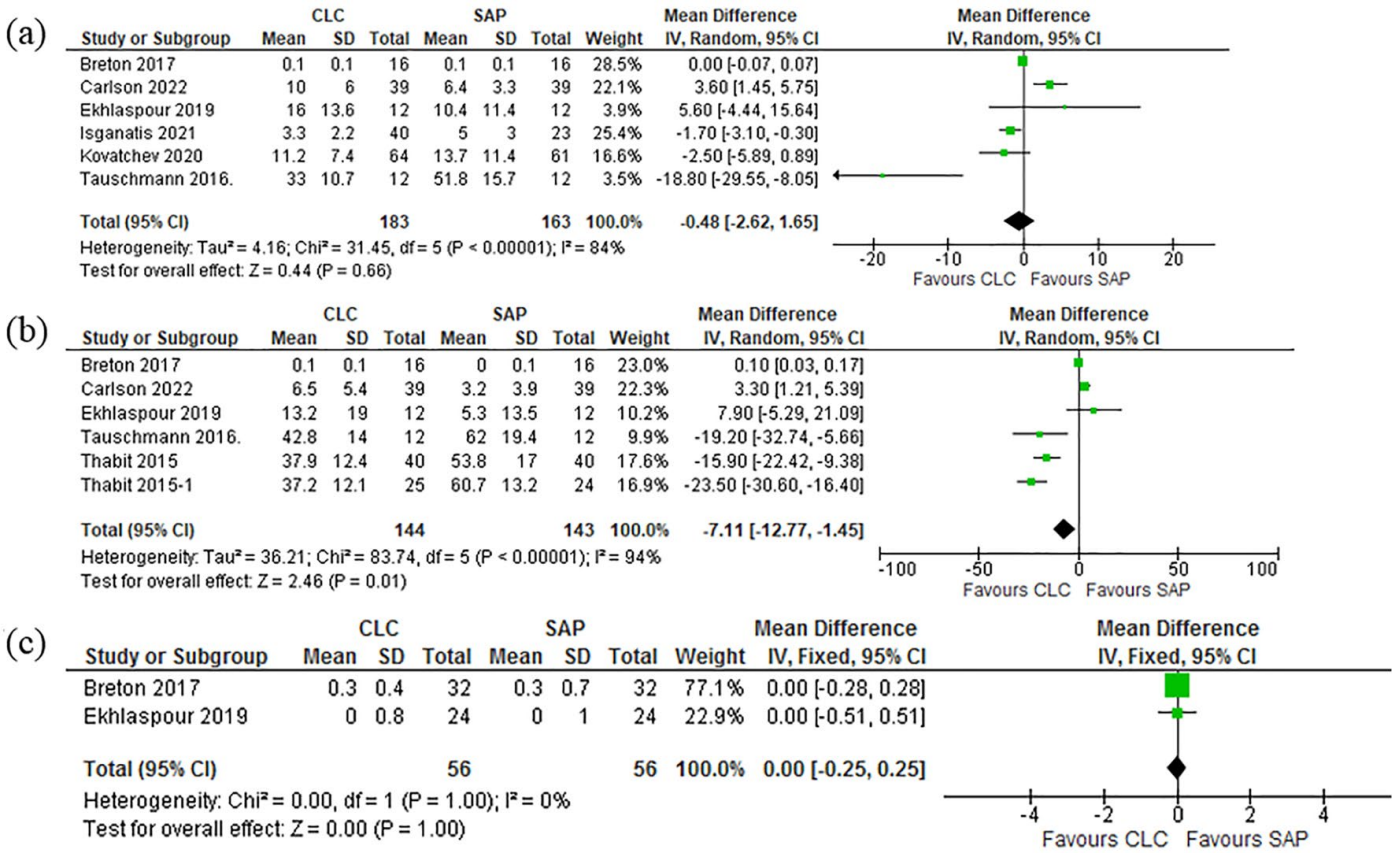
(**a**) Forest plot of comparison: Hyperglycemia-Day. (**b**) Forest plot of comparison: Hyperglycemia-Night. (**c**) Forest plot of comparison: Hyperglycemia-Physical activity.

Hyperglycemic events were monitored to assess the AEs of both the insulin delivery systems under comparison. Six studies^37–41,43^ reported day time results for hyperglycemic events and the pooled results are [Mean Difference (IV, Random, 95% CI) − 0.48 [− 2.62, 1.65]]. Pooled studies show [Heterogeneity: Tau^2^ = 4.16; Chi^2^ = 31.45, df = 5 (P < 0.00001); I^2^ = 84%. Test for overall effect: Z = 0.44 (P = 0.66)]. From the six studies^37–41,43^ which reported the night comparison results for hyperglycemia were pooled and the results shows [Mean Difference (IV, Random, 95% CI) − 7.11 [− 12.77, − 1.45]]. Pooled studies show [Heterogeneity: Tau^2^ = 36.21; Chi^2^ = 83.74, df = 5 (P < 0.00001); I^2^ = 94%. Test for overall effect: Z = 2.46 (P = 0.01)]. Hyperglycemia during physical activities were reported by two studies^37,39^ which were pooled in this meta-analysis and the results are as follows, [Mean Difference (IV, Random, 95% CI) − 0.00 [− 0.10, 0.10]] Pooled studies show [Heterogeneity: Chi^2^ = 3.45, df = 1 (P = 0.06); I^2^ = 71%. Test for overall effect: Z = 0.04 (P = 0.97)]. Forest plot in Fig. 7a–c are illustrative of these pooled results.

## Discussion

A significant morbidity and mortality have been associated with T1DM among adolescents due to its poor prognosis. Before, SAP therapy was a crucial advancement in diabetes treatment but, currently the development of CLC insulin delivery systems has been dramatically gaining clinical importance. Till now, there has been no comprehensive meta-analyses comparing the day and night time efficacy and safety features between traditional SAP therapy and the currently used CLC insulin delivery for pediatric and adolescents with type 1 diabetes. This study is the first meta-analysis to examine the day and night time efficacy and safety monitoring comparison of CLC insulin delivery systems versus SAP therapy in the treatment of adolescents with T1DM. The results from this study shows the supreme authority of the CLC insulin delivery systems in maintaining all the glycemic outcomes than SAP therapy for day and night values of mean BG, TIR, and SD of glucose variability. This study considered the monitoring results during daily routine activities along with strenuous physical activities like skiing and camp activities. Thus, this study, clearly updates and upholds well-defined evidence of the CLC insulin delivery’s efficacy to be clinically used for young patients with T1DM. The safety comparison results from this studies identifies that, CLC insulin delivery was associated with fewer AEs, especially hypoglycemic and hyperglycemic-related events during day and night, than SAP which deems CLC to be an ideal treatment of choice for adolescents with type 1 diabetes.

## Limitations

The limitation of this study is that only 11 RCTs were included, and the number of included patients were limited. One^45^ among the included studies though it participated adolescent patients, failed to separate the adolescent result reports from the adults. Many studies and RCTs are concentrated around the insulin therapy of adults and pediatric T1DM based studies are not given enough attention. Although in this study, CLC insulin delivery was seen to have decreased risk of AEs, hypoglycemia is still the dominant AE. There is an imminent need of extensive high-quality RCTs to ensure the reliability of this conclusion. The included studies were mostly conducted in Europe and United States, which may cause a regional bias in identifying with race related outcomes. There was significant heterogeneity when all the included studies were pooled, which could decrease the reliability of the results. The included studies used different equipments, which possibly increased the heterogeneity of the results. This led to the exclusion of studies with conflicting results from the majority of included studies and had more confidence results. For this reason, random-effects meta-analysis was utilized to incorporate heterogeneity among studies. Finally, all the 11^37–47^ included studies had the industrial support on their research which can have potential influence results reported by them, favoring the equipment or technology used in the experiment.

## Conclusion

CLC insulin delivery exhibits significantly better day and night efficacy and safety than SAP therapy in adolescents with type 1 diabetes. Closed-loop safely and significantly improves glycemic control, maintains time in range, reduces hypoglycemia and hyperglycemia in adolescent populations with T1DM. Tedious and continuous technical improvement of closed-loop systems is required to further improve safety and efficacy, likely through the development of open-frame, personalized, cloud-based ecosystems.

## Data Availability

The author confirm that the data supporting the findings of this study are available within the article.
